# 

**Published:** 1896-05

**Authors:** 


### BOOKS RECEIVED.

Diagnosis and Treatment of Diseases of the Rectum, Anus and Contiguous Textures. Designed for Practitioners and Students. By S. G. Gant, M. D., Professor of Diseases of the Rectum and Anus, University and Woman's Medical Colleges; Rectal and Anal Surgeon to All-Saints, German and Scarritt's Hospital for Women, Kansas City, etc. With two chapters on Cancer and Celiotomy, by Herbert William Allingham, F. R. C. S., Eng., Surgeon to the Great Northern Hospital, etc. Octavo, pp. xiv.—399. Illustrated with sixteen full-page chromo-lithographic plates and 115 wood-engravings in the text. Philadelphia: F. A. Davis Co. 1896.

Transactions of the Medical Society of the State of North Carolina. Forty-second Annual Meeting, held at Goldsboro, N. C., May 14, 15 and 16, 1895. Wilmington, N. C.: Ledwin Brothers, Printers and Binders. 1895.

An Inquiry into the Difficulties Encountered in the Reduction of Dislocations of the Hip. By Oscar H. Allis, M. D., Fellow of the College of Physicians and of the Academy of Surgery, Philadelphia, etc. The Samuel D. Gross Prize Essay. Philadelphia. 1896.

Coca and Its Therapeutic Application. By Angelo Mariani. With illustrations. Third edition. New York: J. N. Jaros, 52 W. 15th Street. 1896.

Transactions of the First Pan-American Medical Congress, held in the City of Washington, D. C., U. S. A., September 5, 6, 7 and 8, 1893. In two volumes. Edited by Charles A. L. Reed, M. D., Secretary-General, Cincinnati, O. Washington: Government Printing Office. 1895.

Diets for Infants and Children in Health and Disease. By Louis Starr, M. D., Editor American Text-book of the Diseases of Children. Price, $1.25 net. Philadelphia: W. B. Saunders, 925 Walnut Street. 1896.

Dame Fortune Smiled: The Doctor's Story. By Willis Barnes. Cloth, $1.25; paper, 50 cents. Boston, Mass.: The Arena Publishing Company, Copley Square.

Voice Building and Tone Placing, Showing a New Method of Relieving Injured Vocal Cords by Tone Exercises. By H. Holbrook Curtis, Ph. B., M. D. Fellow of the New York Academy of Medicine; Fellow of the American Laryngological, Rhinological and Otological Association, etc. Pp. 227. Illustrated. New York: D. Appleton & Co. 1896.

The National Dispensatory with Supplement embracing the new edition of the National Formulary.—The National Dispensatory. Containing the Natural History, Chemistry, Pharmacy, Actions and Uses of Medicines, including those recognised in the Pharmacopeias of the United States, Great Britain and Germany, with numerous references to the French Codex. By Alfred Stille, M. D., LL. D., Professor Emeritus of the Theory and Practice of Medicine and of Clinical Medicine in the University of Pennsylvania; John M. Maisch, Phar. D., late Professor of Materia Medica and Botany in Philadelphia College of Pharmacy, Secretary to the American Pharmaceutical Association; Chas. Caspari, Jr., Ph. G., Professor of Pharmacy in the Maryland College of Pharmacy, Baltimore, and Henry C. C. Maisch, Ph. G., Ph. D. Fifth edition, thoroughly revised in accordance with the new U. S. Pharmacopeia (seventh decennial revision) and embracing the new edition of The National Formulary. In one magnificent imperial octavo volume of 2025 pages, with 320 engravings. Cloth, $7.25; leather, $8.00. With Ready Reference Thumb-letter Index, cloth, $7.75; leather, $8.50. Philadelphia and New York: Lea Brothers & Co., Publishers. 1896.

International Clinics. A Quarterly of Clinical Lectures on Medicine, Neurology, Surgery, Genito-urinary Surgery, Gynecology, Pharyngology, Rhinology, Ophthalmology, Laryngology, Obstetrics, Otology and Dermatology. By professors and lecturers in the leading medical colleges of the United States, Great Britain and Canada. Edited by Judson Daland, M. D. (Univ. of Penna.), Philadelphia, Instructor in Clinical Medicine and Lecturer on Physical Diagnosis in the University of Pennsylvania; Assistant Physician to the University Hospital; Physician to the Philadelphia Hospital and Fellow College of Physicians, Philadelphia. J. Mitchell Bruce, M. D., F. R. C. P., London, England, Physician to, and Lecturer on, the Principles and Practice of Medicine at the Charing Cross Hospital. David W. Finlay, M. D., F. R. C. P., Aberdeen, Scotland, Professor of Practice of Medicine in the University of Aberdeen; Physician to, and Lecturer on, Clinical Medicine in the Aberdeen Royal Infirmary; Consulting Physician to the Royal Hospital for Diseases of the Chest, London. Volume IV. Fifth series. 1896. Royal 8vo, pp. x.—363. Philadelphia: J. B. Lippincott Co. 1895.

Tenth Annual Report of the State Board of Health and Vital Statistics of the Commonwealth of Pennsylvania. Benjamin Lee, M. D., Secretary. Philadelphia: Clarence M. Busch, State Printer. 1895.

Fifteenth Annual Report of the State Board of Health of New York. Including eighteen maps. Transmitted to the Legislature March 6, 1895. Albany: James B. Lyon, State Printer. 1895.

